# Supplementary material for: A protein interaction mechanism for suppressing the mechanosensitive Piezo channels
Source: Nat Commun. 2017 Nov 27;8:1797. doi: 10.1038/s41467-017-01712-z (PMC5702604; doi:10.1038/s41467-017-01712-z)
Supplement: Supplementary file 1 — Supplementary Information [file 41467_2017_1712_MOESM1_ESM.pdf]

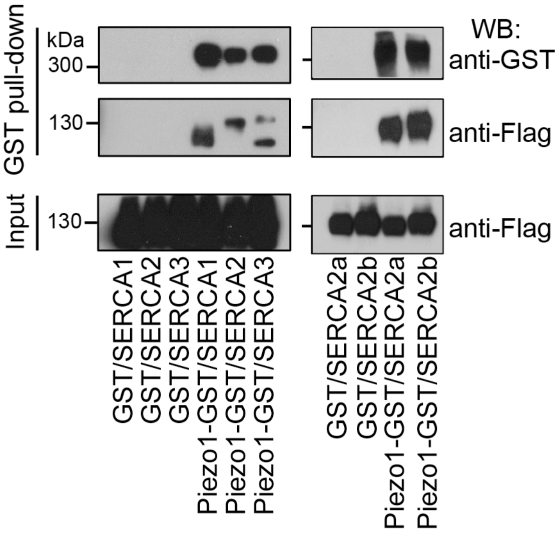

Supplementary Figure 1 The three isoforms of SERCA (SERCA1, 2, 3) and both of the splicing variants of SERCA2 (SERCA2a, 2b) interact with Piezo1

Cell lysates of HEK293T cells transfected with the indicated constructs were subjected to GST pull-down and subsequent western blotting with the anti-GST and anti-Flag antibodies (repeated 3 times).

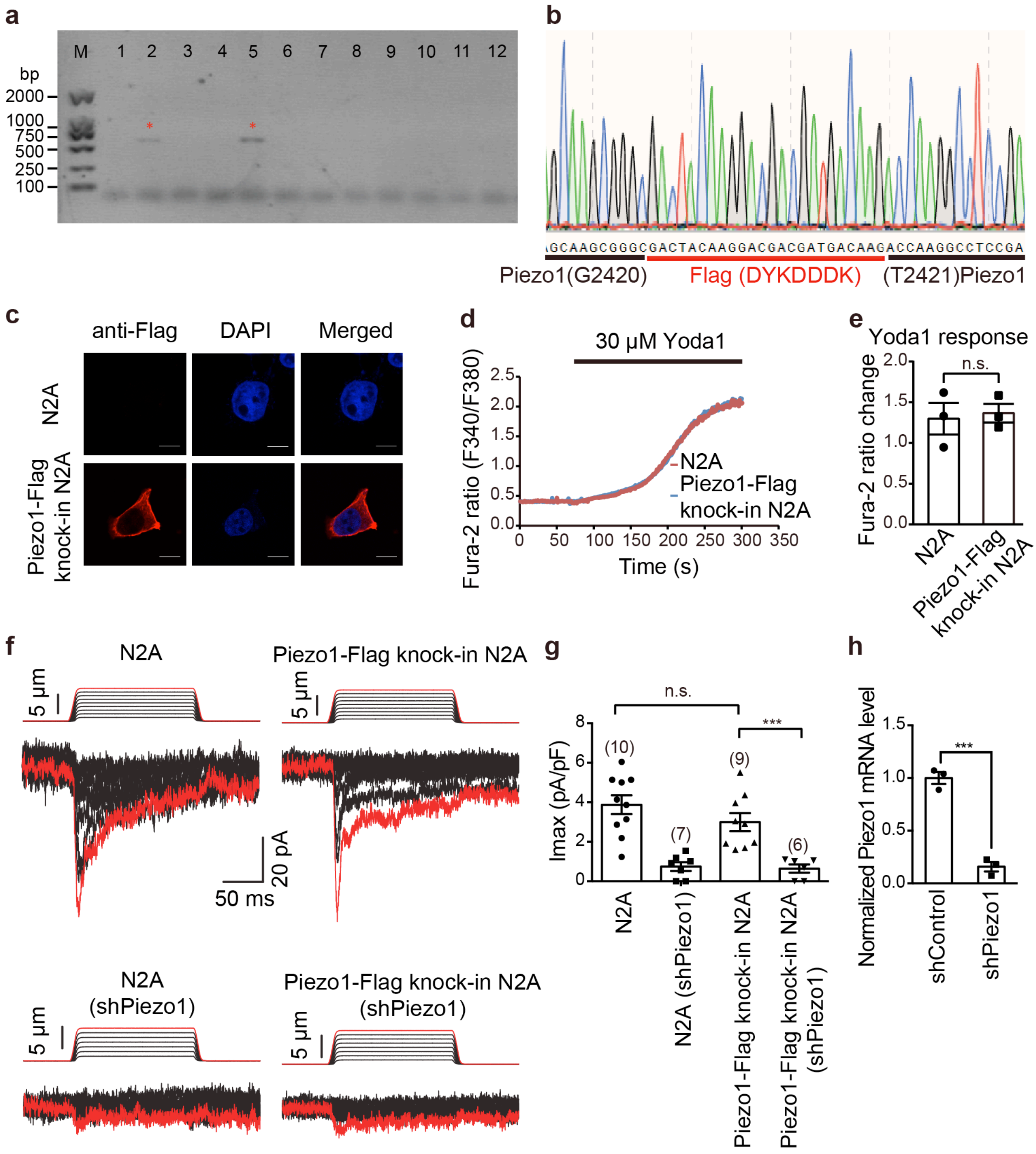

Supplementary Figure 2 Generation and characterizations of the Piezo1-Flag knock-in N2A cells

a, PCR screening of N2A clones after CRISPR-Cas9 targeting. Clones #2 and #5 show the expected PCR product.

b, Sequencing verification of the correct insertion of the Flag-encoding sequence.

c, Immunofluorescent staining of wild-type or Piezo1-Flag knock-in N2A cells with the anti-Flag

antibody. Scale bar, 10 μm.

d, Representative average traces of single-cell Fura-2 Ca^{2+} imaging of the indicated cells in response to the Piezo1 chemical activator Yoda1.

e, Scatter plots of the Yoda1-induced Fura-2 ratio amplitude changes of the indicated cells. Unpaired student's t-test.

f, Representative traces of poking-induced inward currents recorded at -60 mV in wild type N2A or Piezo1-Flag knock-in N2A cells with or without knocking down Piezo1 with the shRNA against Piezo1 (shPiezo1).

g, Scatter plots of poking-induced maximal currents of the indicated conditions. One-way ANOVA with multiple comparison test.

h, Quantitative Real-Time PCR analysis of Piezo1 mRNA in N2A cells for validating the knockdown efficiency of Piezo1 shRNA. Glyceraldehyde-3-phosphate dehydrogenase (GAPDH) was used as the reference gene by means of the $2^{-\Delta\Delta CT}$ method. Unpaired student's t-test. $^{***}p < 0.001$.

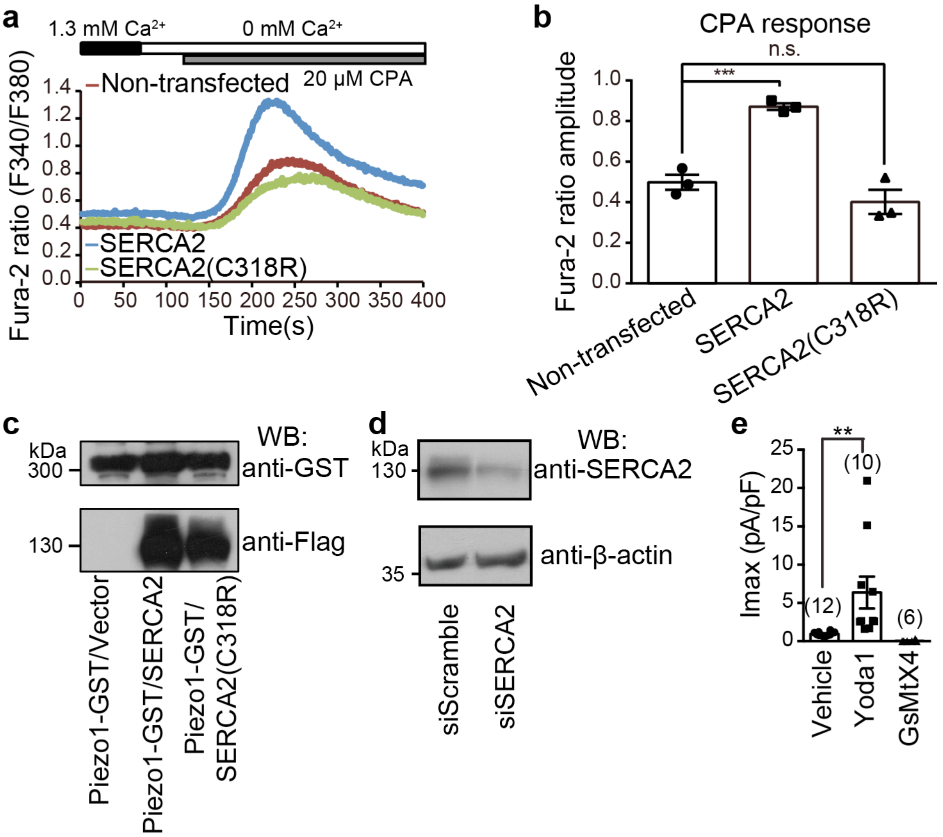

Supplementary Figure 3 Characterizations of the SERCA2 (C318) mutant that lacks Ca^{2+} pumping activity, the knockdown efficiency of SERCA2 in N2A, and pharmacological responses of poking-induced currents in HUVEC.

a, Representative average traces of single-cell Fura-2 Ca^{2+} imaging of HEK293T cells transfected with the indicated constructs in response to cyclopiazonic acid (CPA), an inhibitor of SERCA2.

b, Scatter plots of the CPA-induced Fura-2 ratio amplitude changes of the indicated cells. One-way ANOVA with Turkey's multiple comparison test.

c, Western blotting detection of the expression of Piezo1, SERCA2 and SERCA2-C318R in HEK293T cells co-transfected Piezo1-GST with vector, Flag-SERCA2 or Flag-SERCA2-C318R.

d, Western blotting shows the knockdown efficiency of SERCA2 in N2A cells (repeated 3 times).

e, Scatter plots of the poking-induced maximal currents from HUVEC treated with Vehicle, 30 μM Yoda1 and 4 μM GsMTX4. One-way ANOVA with Turkey's multiple comparison test. $^{**}p < 0.01$, $^{***}p < 0.001$.

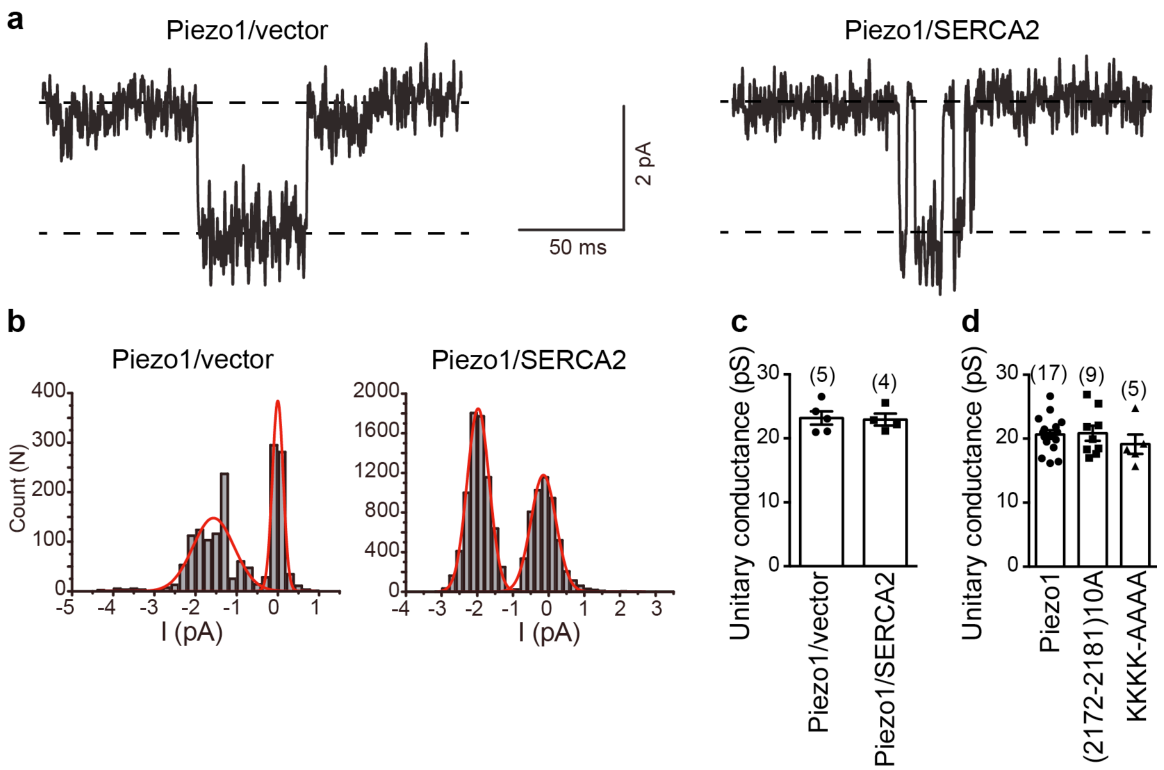

Supplementary Figure 4 SERCA2 co-expression or mutating the linker region does not affect the unitary conductance of Piezo1.

a, Representative traces showing single-channel activities of cells with the indicated transfections in the absence of mechanical stimulation.

b, All-event current amplitude histograms of the recordings. Current amplitudes recorded at -80 mV were calculated from Gaussian fits to the current amplitude histograms. 150 to 1400 or 40 to 1800 events were analyzed for individual Piezo1/Vector- (5 cells) or Piezo1/SERCA2-transfected (4 cells) cells, respectively.

c and **d**, Scatter plots of the unitary conductance of the indicated transfections.

The analyzed cell number is labeled above the bar.

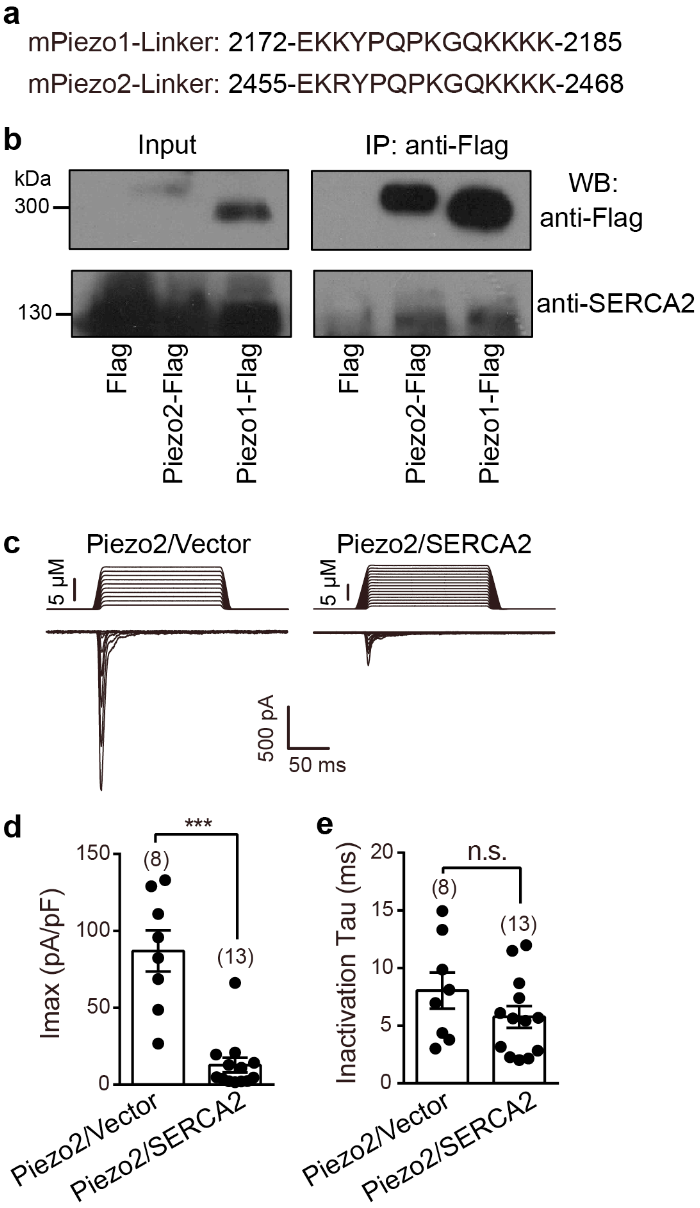

Supplementary Figure 5 SERCA2 interacts with Piezo2 and suppresses its poking-evoked currents.

a, Sequence conservation of the linker region between mPiezo1 and mPiezo2.

b, Western blots showing co-precipitation of SERCA2 with either Piezo1 or Piezo2.

c, Representative traces of poking-induced currents recorded at -60 mV in HEK293T cells with the indicated transfections.

d and **e**, Scatter plots of the maximal poking-induced currents (**d**) or inactivation tau (**e**) of the indicated transfections. Unpaired student's t-test. $^{***}p < 0.001$

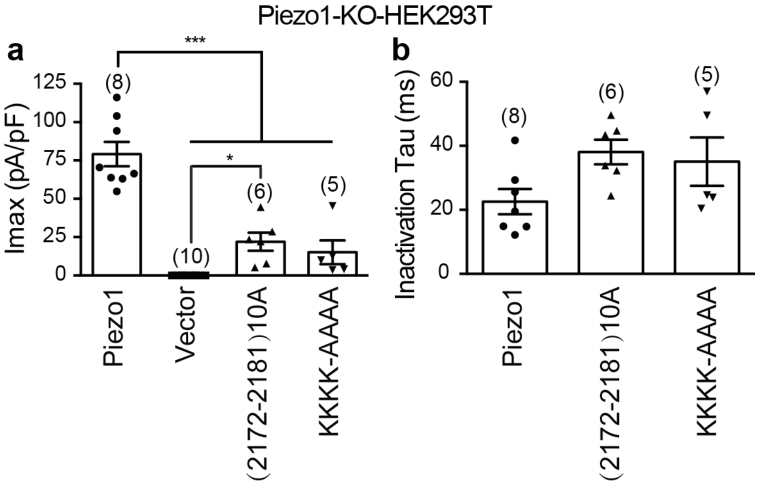

Supplementary Figure 6 Piezo1-KO-HEK293T cells transfected with the Piezo1-(2172-2181)10A and Piezo1-KKKK-AAAA show reduced poking-induced currents compared to Piezo1-transfected cells, similar to that observed in HEK293T cells shown in Figure 5d - f.

a and **b**, Scatter plots of the maximal poking-induced currents (**a**) or inactivation tau (**b**) recorded from the Piezo1-KO-HEK293T cells transfected with the indicated constructs. One-way ANOVA with multiple comparison test. $^{***}p < 0.001$, $^{*}p < 0.05$.

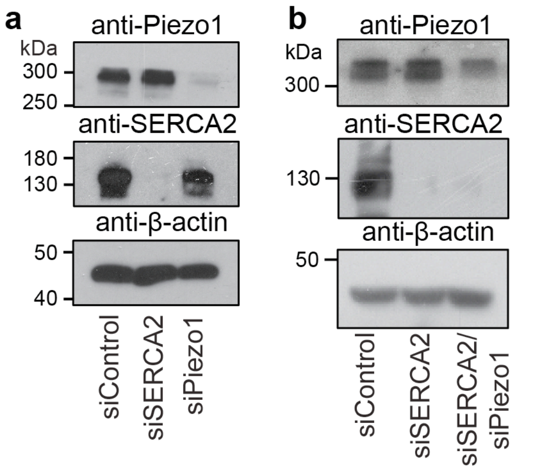

Supplementary Figure 7 Knock-down efficiency of SERCA2 and Piezo1 in HUVEC.

a and **b**, Western blotting shows the knockdown efficiency of SERCA2 and Piezo1 (repeated 3 times).

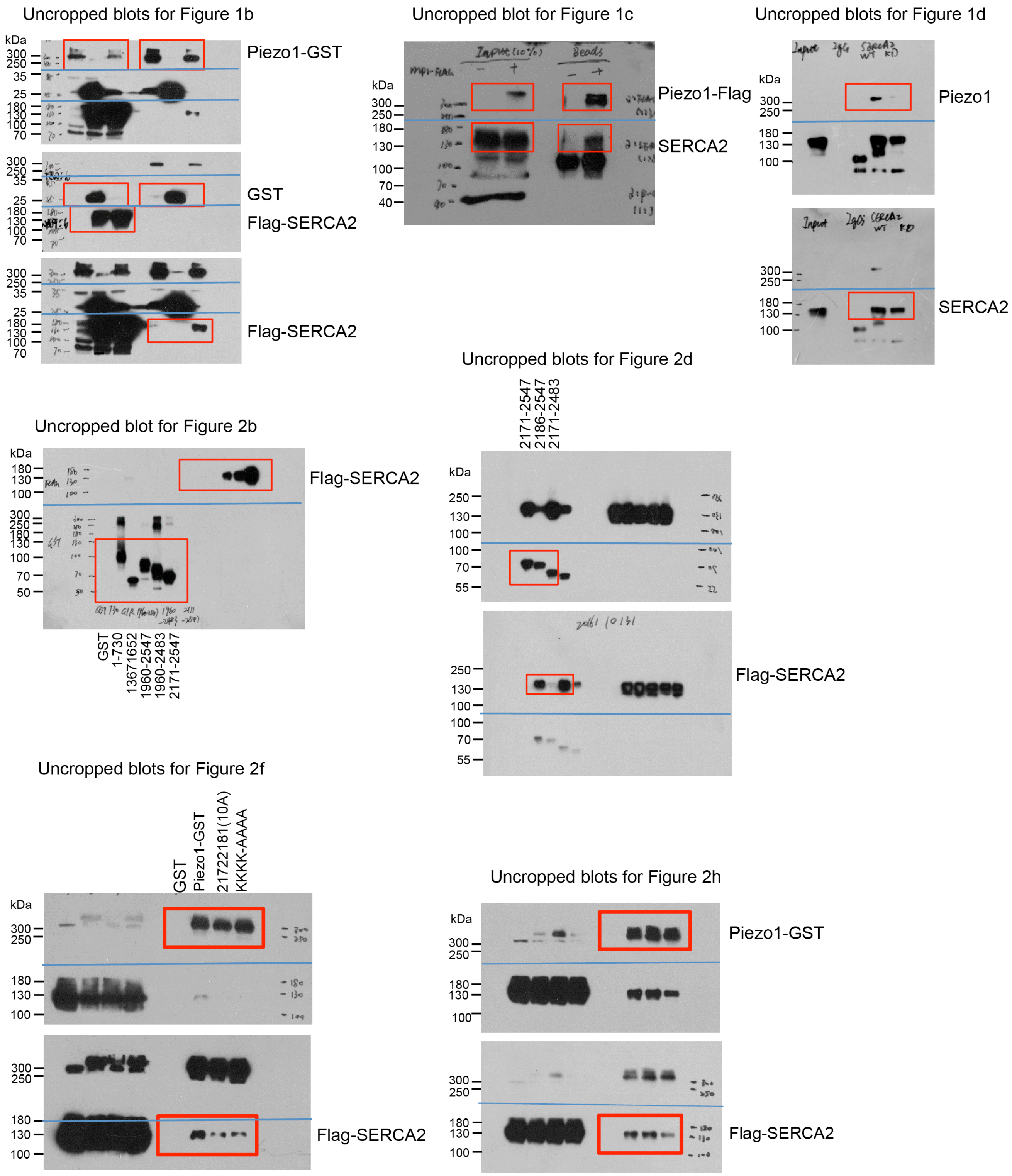

Supplementary Figure 8 Uncropped western blots

Red boxes in the uncropped blots indicate the cropped regions shown in the corresponding figures.

Supplementary Fig. 8 (continued)

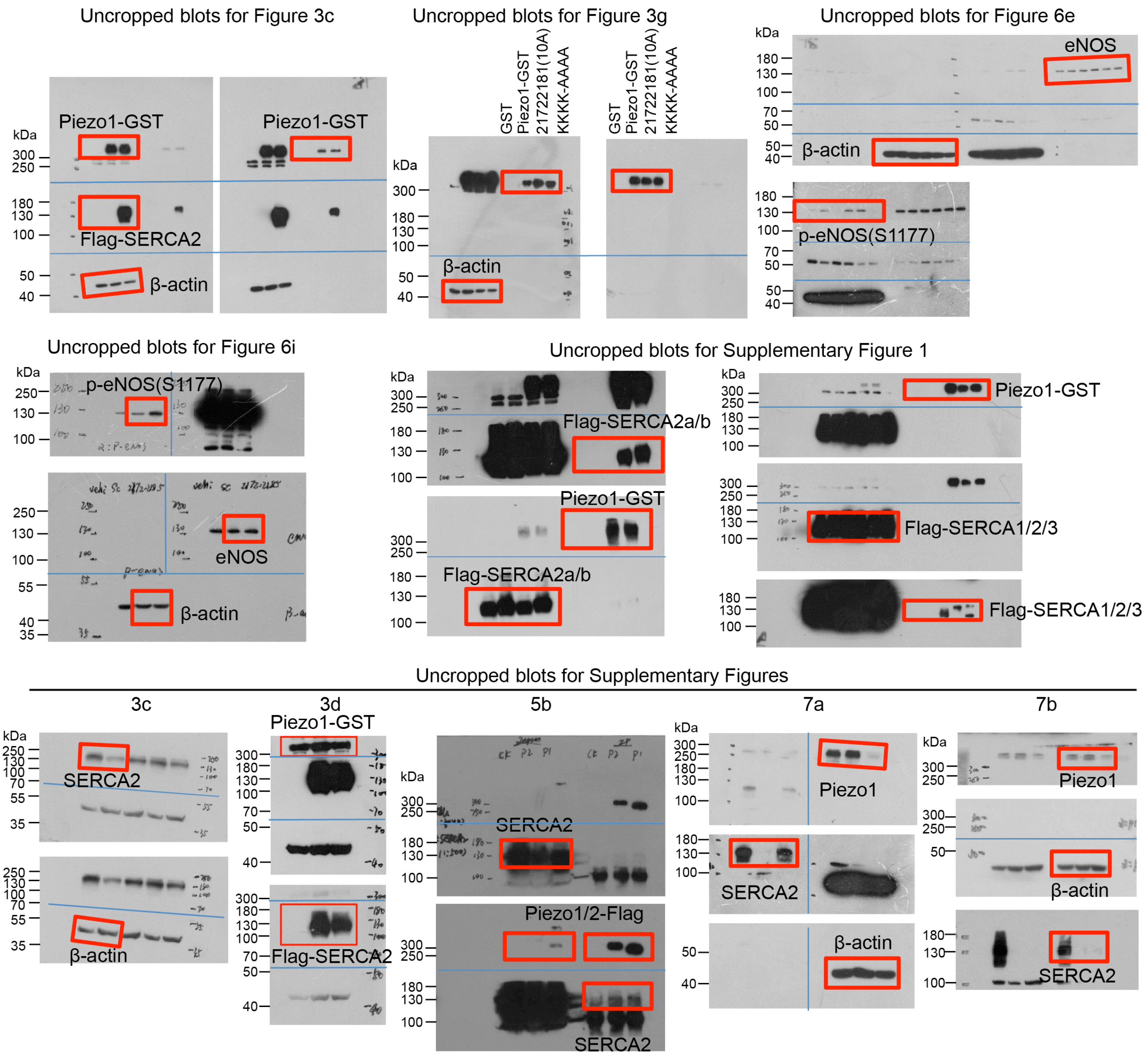

Supplementary Table 1 Peptides identified by mass spectrometry that correspond to SERCA2

Sequence	# PSMs	# Proteins	# Protein Groups	Protein Group Accessions	Modifications	ΔCn	q-Value	PEP	XCorr	Charge	MH+ [Da]	ΔM [ppm]	RT [min]
IRDEMVATEQER	2	5	1	J3KMM5(SERCA2)		0	0	0.002756	3.49	2	1476.7158	3.08	20.22
VGEATETALTcLVEK	3	14	2	P13585(SERCA1);J3KMM5	C11(Carbamidomethyl)	0	0	3.39E-06	3.43	2	1620.814	-0.61	31.6
NMLFSGTNIAAGK	1	6	1	J3KMM5		0	0	0.0002192	3.28	2	1323.6719	-0.53	29.61
MNVFDTELKGLSK	1	5	1	J3KMM5		0	0	0.0002284	3.16	2	1481.767	0.06	32.5
VDQSILTGESVSVIK	2	7	2	P13585;J3KMM5		0	0	1.81E-06	3.15	2	1574.8641	0.26	29.82
NmLFSGTNIAAGK	1	6	1	J3KMM5	M2(Oxidation)	0	0	0.00785	2.68	2	1339.6672	-0.28	26.69
SMSVYcTPNKPSR	1	5	1	J3KMM5	C6(Carbamidomethyl)	0	0	0.005171	2.55	2	1526.7103	0.78	20.16
TGTLTTNQMSVcR	1	13	1	J3KMM5	C12(Carbamidomethyl)	0	0	0.000374	2.41	2	1468.6889	0.38	21.93
IGIFGQDEDVTSK	1	4	1	J3KMM5		0	0	0.0001098	2.28	2	1408.6957	0.15	29.08
EFDELSPSAQR	2	4	1	J3KMM5		0	0	0.007019	2.16	2	1278.5964	0.18	23.77
ANAcNSVIK	1	6	2	P13585;J3KMM5	C4(Carbamidomethyl)	0	0	0.0009914	2.12	2	976.48802	-0.06	17.03
GAPEGVIDR	2	7	2	P13585;J3KMM5		0	0.003	0.01901	2.01	2	913.4735	-0.37	19.15
EWGSGSDTLR	1	5	1	J3KMM5		0	0.003	0.02619	1.91	2	1107.5069	0.27	22.9
SEIGIAMGSGTAVAK	1	4	1	J3KMM5		0	0.005	0.03448	2.51	2	1391.7204	0.29	26.21
RIGIFGQDEDVTSK	1	4	1	J3KMM5		0	0.007	0.03952	3.03	2	1564.7956	-0.68	27.44
LDEFGEQLSK	1	7	2	P13585;J3KMM5		0	0.007	0.05005	2.17	2	1165.5727	-0.79	25.6

Supplementary Table 2 Primer, sgRNA, shRNA and siRNA sequences

Name	Sequence
mPiezo1-F	GCCCTCTAGACTCGAGCGGCCGCGCCACCATGGA
mPiezo1-SbfI-F	CCTGAAAGCCACAGCCCTGCAGG
mPiezo1-R	AACAGAACTTCCAGTGGCGCGCCAAGCTTCTTCT
mPiezo1-Flag-R	AAGGTTCCGCGGCTACTTATCGTCGTCATCCTTGTAATCTGG CGCGCCAAGC
mPiezo1-730-R	AACAGAACTTCCAGTGGCGCGCCAAGCTTCCATCGAGGGTGGCGGGTG
mPiezo1-1367-F	GCCCTCTAGACTCGAGCGGCCGCGCCACCATG
mPiezo1-1652-R	AACAGAACTTCCAGTGGCGCGCCAAGCTTCC
mPiezo1-1960-F	CGTTTAAACGGGCCCTCTAGAGCCACCATGCGTTTCTT
mPiezo1-2171-F	CGTTTAAACGGGCCCTCTAGAGCCACCATGACAGAGAAGAAATACCCC
mPiezo1-2186-F	CGTTTAAACGGGCCCTCTAGAGCCACCATGATTGTCAAGTATGGTATG
mPiezo1-2483-R	AACAGAACTTCCAGTGGCGCGCCAAGCTTGCCCCGCACAAACTTGCC
mPiezo1-2172-2181A-F	AGCCGCTGCGGCAGCCGCAGCGGCCAAGAAGAAGAAAATTGTCA
mPiezo1-2172-2181A-R	CTGCGGCTGCCGCAGCGGCTGCAGCTGTCTCTCGGCTGCACTTG
mPiezo1-KKKKAAAA-F	GCAGGCTGCCGCAGCGATTGTCAAGTATGGTATGGG
mPiezo1-KKKKAAAA-R	CAATCGCTGCGGCAGCCTGCCCCTTGGGCTGGGGGT
mPiezo1-mRuby-F	AGGGAGAAGAAGCTTGGCGCGCCAATGGTGTCTAAGGGCGAA
mPiezo1-mRuby-R	TTTAAACTTAAGCTTGGCCGGCCTCACTTGTCATCGTCGTCCTTGTA GTCTCCATCGAGTGATCCCTTGTACAGCTCGTCCAT
mPiezo2-6684KasI-F	TGTGAGGCGCCGCCCTCT
mPiezo2-Flag-R	GAT GCGGCCGCCTACTTATCGTCGTCATCCTTGTAATC TGGCGCGCCTCCG
Flag-SERCA2-F	AGCTCCACCGCGGTGGCGGCCGCCACCA
Flag-SERCA2-R	GGGCCCCCCCTCGAGGTCGACTCAA
Flag-SERCA2-IRES-GFP-F	GCCCTCTAGACTCGAGCGGCCGCCACCATGGATTA
Flag-SERCA2-IRES-GFP-R	GGGCGGATCCCGGGCCCGCGGTCAAGACCAGAACATATCGC
SERCA2-C318R-F	GCAGTCATCACCACCCGCCTGGCTCTTGGAA
SERCA2-C318R-R	AGGCGGGTGGTGATGACTGCAGGCA
SpeI-SERCA2-F	GGTGAATTCCTCGAGACTAGTGCCACCATGGAGAACGCGCACAC
NotI-SERCA2-R	GAGAGGGGCGGATCCGCGGCCGCTCAAGACCAGAACATATC
FIag-SERCA2a-R	GGGCCCCCCCTCGAGGTCGACTTACTCCAGTATTGCA GGTTCCAGGTAGTTGCG
Flag-SERCA1-F	GATAAGAGCCCGGGCGGATCCATGGAGGCCGCGCACTCCAAGT
Flag-SERCA1-R	GGGCCCCCCCTCGAGGTCGACTTATCCCTCCAGATAGTTCCGA
Flag-SERCA3-F	GATAAGAGCCCGGGCGGATCCATGGAGGAGGCGCACCTGCTCT
Flag-SERCA3-R	GGGCCCCCCCTCGAGGTCGACTCAGTCTGAGGGCCACACTGGA
Flag-pCMV-Tag2b-F	CCGGGCGGATCCTGAGAATTCG
Flag-pCMV-Tag2b-R	TCGACGAATTCTCAGGATCCGC
Flag-pcDNA3.1(-)-F	GGCCGCACTCGAG ATGGACTACAAAGACGATGATGACAAGTAG CCGC
Flag-pcDNA3.1(-)-R	GG CTACTTGTCATCATCGTCTTTGTAGTCCATCTCGAGT GC
IRES-GFP-F	GACGACGATAAGTAGGCGGCCGCTTAACTGCAGTCGA
IRES-GFP-R	GCCCTCTAGACTCGAGCGGCCGCCGGTTTAAACTTAA
GST-F	CTAGACTCGAGCGGCCGCGCCACCATGAAGCTTGG
GST-R	CGCGCCAAGCTTCATGGTGGCGCGGCCGCTCGAGT

Supplementary Table 2 (continued)

Name	Sequence
mPiezo1 shRNA-F	AACTCGGCGCTTGCTAGAACTTCATTCAAGAGATGAAGTTCT AGCAAGCGCCGATTTTC
mPiezo1 shRNA-R	TCGAGAAAATCGGCGCTTGCTAGAACTTCATCTCTTGAATGAA GTTCTAGCAAGCGCCGAGTT
mPiezo1-qPCR-F	TGCCATGCTCCTCTATCTGCT
mPiezo1-qPCR-R	GGCGCACACATAGATCCAGTA
mGAPDH-qPCR-F	GCACCACCAACTGCTTAG
mGAPDH-qPCR-R	GGATGCAGGGATGATGTTC
mPiezo1-Flag sgRNA-F	CACCGTGGGGAGCAAGCGGGCACCA
mPiezo1-Flag sgRNA-R	AAACTGGTGCCCGCTTGCTCCCCAC
mPiezo1-Flag donor1-F	CGTTTAAACGGGCCCTCTAGACACACTGCCTAACACTGCCTGC
mPiezo1-Flag donor1-R	CTTGTCATCGTCGTCCTTGTAGTCGCCCGCTTGCTCCCCAGA
mPiezo1-Flag donor2-F	ACAAGGACGACGATGACAAGACCAAGGCCTCCGACTTCCTC
mPiezo1-Flag donor2-R	TAGTCCAGTGTGGTGGAATTCAGGCAGCTCCTTCATTCCCG
mPiezo1 shRNA	UCGGCGCUUGCUAGAACUUCA
Control (Scrambled) siRNA	UUCUCCGAACGUGUCACGU
hPiezo1 siRNA	AGAAGAAGAUCGUCAAGUA
hSERCA2 siRNA mix pool	AAGCAGGACAUCAAUGAGCAA
	AAGGUGAUACUUGUUCCCUUA
	CAACUGGAGUUAACACCGAAA
	CAGAAAGUCAAUGUCGGUUUA

Supplementary Table 3 Sequence information for generating the Piezo1-Flag knock-in N2A

Name	Sequence
mPiezo1 sgRNA	UGGGGAGCAAGCGGGCACCA
Insert sequence of the donor plasmid	CACACTGCCTAACACTGCCTGCTCCCCAGGGACCTGGCCAAGGGTGGCACTGTG<br>GAGTATACTAATGAGAAGCACACCTTGGAGCTGGCCCCCAACAGTACGGCACGA<br>AGGCAGCTGGCCCAACTGCTCGAGGGCAGACCTGACCAGTCAGTGTGAGTGGGG<br>GCCACTGTGGGGCATGTGGGCAGGAGGGCTAAGATGCCCCTGACCTGAGCAGTC<br>AGTGTGAGTGGGGGCCACTGTGGGGCATGTGGGCAGGAGGGCTAAGATGCCCCT<br>GACTTGCTGACTCCTCCCACAGGGTCATTCCCCATCTCTTCCCCAAGTACATTCGT<br>GCTCCCAATGGGCCTGAAGCCAACCCTGTGAAGCAGCTGCAGCCAGGTCAGTGT<br>GGGAGCAGGGCGCAGGGATGTGGGCTAGGCCGGAAGTGTGGGTGCAGGGATGT<br>AGGGATGCGGGCTAGGCCGGGGGCCAGTGTAGCCCGACTCTAACTATCCCACTC<br>AACTCAGATGAGGAAGAGGACTACCTTGGTGTGCGCATCCAGCTGCGGAGGGAG<br>CAAGTGGGCACAGGGGCCTCTGGGGAGCAAGCGGGCGACTACAAGGACGACGA<br>TGACAAGACCAAGGCCTCCGACTTCCTCGAGTGGTGGGTCATCGAGCTGCAGGA<br>CTGCAAGGCTGACTGCAACCTGCTGCCCATGGTCATCTTCAGTGACAAGGTCAGC<br>CCACCTAGCCTGGGCTTCCTGGCCGGCTACGGGTGAGTACTGCAGAGGGATCTCC<br>GAGAGCCCCAAGGGTTCTGAGTGGTCCCGGCTCACATGCCCGCCTCTCCCGACA<br>GGATTGTGGGGCTGTACGTCTCCATCGTGCTGGTGGTTGGCAAGTTTGTGCGGGG<br>CTTCTTCAGCGAGATCTCTCACTCCATCATGTTCGAGGAACTGCCGTGTGTGGAC<br>CGCATCCTCAAGCTGTGCCAGGACATCTTCTTGGTGCGCGAGACCCGGGAGCTG<br>GAGCTGGAGGAGGAGCTATACGCCAAGCTCATCTTCCTGTACCGATCTCCAGAG<br>ACCATGATTAAGTGGACACGTGAGAGGGAGTAGGAGCCCAGGCCCTGGGCACCG<br>GGAATGAAGGAGCTGCCT
