# Supplementary material for: A protein interaction mechanism for suppressing the mechanosensitive Piezo channels
Source: Nat Commun. 2017 Nov 27;8:1797. doi: 10.1038/s41467-017-01712-z (PMC5702604; doi:10.1038/s41467-017-01712-z)
Supplement: Supplementary file 3 — Description of Additional Supplementary Files [file 41467_2017_1712_MOESM3_ESM.pdf]

Description of Additional Supplementary Files

File Name: Supplementary Data 1
Description: The proteins identified in the mass spectrometry analysis of the bands near 130kDa.
